# Potential Role of Quercetin in Polycystic Ovary Syndrome and Its Complications: A Review

**DOI:** 10.3390/molecules27144476

**Published:** 2022-07-13

**Authors:** Tong Chen, Fan Jia, Yue Yu, Wufan Zhang, Chaoying Wang, Shiqin Zhu, Nana Zhang, Xinmin Liu

**Affiliations:** 1 Department of Gynecology, Guang’anmen Hospital, China Academy of Chinese Medical Sciences, Beijing 100053, China; chentong618@sohu.com (T.C.); jiafan19961120@163.com (F.J.); yuyuemg@126.com (Y.Y.); zwf163001@163.com (W.Z.); tom18811358036@163.com (C.W.); m1881358078@163.com (S.Z.); 15011266878@163.com (N.Z.); 2 China Academy of Chinese Medical Sciences, Beijing 100700, China; 3 Department of Gynecology of Traditional Chinese Medicine, Graduate School, Beijing University of Chinese Medicine, Beijing 100029, China

**Keywords:** quercetin, antioxidant, polycystic ovary syndrome, mechanism

## Abstract

Polycystic ovary syndrome (PCOS) is a common multisystem disease with reproductive, metabolic and psychological abnormalities. It is characterized by a high prevalence rate in women of childbearing age and highly heterogeneous clinical manifestations, which seriously harm women’s physical and mental health. Quercetin (QUR) is a natural compound of flavonoids found in a variety of foods and medicinal plants. It can intervene with the pathologic process of PCOS from multiple targets and channels and has few adverse reactions. It is mentioned in this review that QUR can improve ovulation disorder, relieve Insulin resistance (IR), reduce androgen, regulate lipid metabolism, regulate gut microbiota and improve vascular endothelial function, which is of great significance in the treatment of PCOS.

## 1. Introduction

Polycystic ovary syndrome (PCOS) is a common multisystem disease with reproductive, metabolic and psychological abnormalities [1], which occurs in more than 8–13% of women of childbearing age worldwide [2], and the prevalence of PCOS in China is about 5.6% [3]. The clinical manifestations of PCOS are highly heterogeneous, mainly characterized by sparse menstruation or amenorrhea, hyperandrogenemia, polycystic change of ovary, accompanied by Insulin resistance (IR), obesity and anxiety and depression to varying degrees [4]. In the early stage, PCOS is often complicated with infertility and adverse pregnancy outcomes, while in the long term, the incidence of endometrial cancer, type 2 diabetes and cardiovascular diseases gradually increases, seriously harming women’s physical and mental health.

So far, there is still no effective cure for PCOS, and symptomatic treatment is mainly used, including lifestyle intervention, menstrual cycle adjustment, androgen reduction and improvement in metabolism. PCOS with ovulatory disorder mainly used second-half cycle progesterone or a combination of short-acting oral contraceptives, but they could not restore PCOS spontaneous ovulation and were prone to relapse after drug withdrawal. Metformin is used to improve PCOS metabolic disorder, but its ovulation rate is low, and there are certain adverse reactions, such as diarrhea, nausea, fatigue, headache, etc. [5], resulting in the reduced compliance of patients. Letrozole and clomiphene are suitable for PCOS patients with fertility needs. Letrozole inhibits estrogen synthesis, and clomiphene competitively binds estrogen receptors, both of which reduce estrogen levels, increase follicle-stimulating hormone (FSH) release, and promote follicular development. This low estrogen status increases the risk of pregnancy loss or multiple pregnancies [6]. Secondly, the continuous stimulation of multiple follicles will induce the occurrence of follicular hyperstimulation syndrome. It mainly manifests as ovarian cystic enlargement, capillary permeability increases and systemic edema, which is one of the critical gynecological diseases. Long-term use of clomiphene also increases the risk of ovarian cancer, but the mechanism is unclear and may be related to increased FSH [7]. It can be seen that the current treatment drugs can only target a certain pathological link of PCOS, and there are certain limitations. Therefore, a multi-target, safe and effective drug is urgently needed to provide a new idea for the treatment of PCOS.

**Figure 1:**
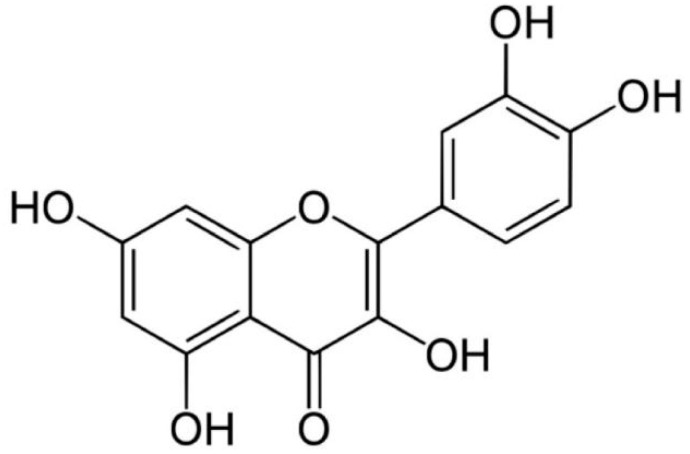
Molecular structure of QUR.

QUR is a natural compound of flavonoids, which is not produced in the human body and is found in a variety of food and medicinal plants, such as apples, onions, grapes, cherries, oranges, tomatoes, ginkgo, forsythia, etc. [8,9]. The chemical structure of QUR is C15H10O7 (Figure 1). QUR is a bitter yellow crystal, completely soluble in alcohol and lipids but insoluble in water [8,10]. After oral administration, about 93.3% of QUR is lost in the gut, and only 3.1% is metabolized in the liver [11]. Studies found no abnormal liver or kidney function after taking QUR, and only a few patients have mild gastrointestinal reactions [12,13]. It has a fast metabolic rate and a short half-life in blood, and its bioavailability is low [14]. Modern pharmacological studies have shown that QUR has anti-oxidation, anti-cancer, anti-allergy, anti-inflammation, anti-obesity and neuroprotection properties and shows an inhibition of platelet aggregation, enhancement of mitochondrial synthesis, regulation of intestinal microorganisms and other functions [8,9,15]. QUR can intervene in multiple pathological processes of PCOS through multi-target and multi-pathway and is expected to become a new treatment drug for PCOS.

In recent years, a large amount of literature has been accumulated in the field of QUR treatment of PCOS. This review aims to provide theoretical basis and broaden ideas for the treatment of PCOS.

## 2. Retrieval Methods

This review searched studies published in the PubMed, Web of Science or Embase database on the effects of quercetin in the treatment of polycystic ovary syndrome between January 1990 and May 2022. Search terms included “quercetin”, “polycystic ovary syndrome”, “ovulation disorders”, “insulin resistance”, “hyperandrogenemia”, “androgen”, “abnormal lipid metabolism”, “obesity”, “inflammation”, “intestinal flora”, and “vascular endothelial dysfunction”. In addition, references and related records were reviewed.

## 3. QUR in the Treatment of PCOS

QUR is an emerging drug for the treatment of polycystic ovary syndrome. Related animal experiments (Table 1) and clinical trials (Table 2) were mainly reported in the last 5 years. Eight studies reported the effect of quercetin in PCOS animal models, in which the effective dose of quercetin for the treatment of PCOS ranged from 15 mg/kg to 150 mg/kg per day, and the treatment lasted from 15 days to 6 weeks. In patients with PCOS, three studies reported the effect of quercetin; the effective dose was 1000 mg, and the treatment lasted for 6 weeks. Except for estradiol (E2), quercetin had the same trend of improving PCOS-related indexes, which suggests that quercetin is effective in the treatment of PCOS. In addition, none of the studies reported serious adverse events, only mild gastrointestinal discomfort.

However, there are still some drawbacks. Firstly, none of the studies mentioned the source of quercetin. In addition, although QUR has been shown to be effective and safe, a major weakness is its low bioavailability, which greatly limits its bioactivity and health benefits. Innovative drug delivery strategies have been developed; examples include QUR-loaded gel, QUR polymeric Micelle, QUR nanoparticles, Glucan-QUR Conjugate, QUR-loaded mucoadhesive nano-emulsions and so on [10]. However, in the literature studies on the treatment of PCOS in the past five years, QUR administration methods mainly included being dissolved in ethanol, oral administration, capsule preparation, etc., and the administration method of improving the bioavailability of QUR was not adopted, which is still worthy of further study.

## 4. Effect and Mechanism of Quercetin in Polycystic Ovary Syndrome

### 4.1. QUR Can Improve Ovulation Disorders

Ovulation disorder is one of the most common types of PCOS, accounting for about 75% [27], and is the primary cause of infertility associated with PCOS. At present, the mechanism of ovulation disorders is not fully elucidated. It is well-known that follicle development is controlled by the hypothalamic-pituitary-ovarian (HPO) axis, while the abnormal secretion of hormones regulated by this gonadal axis affects follicular development. In addition, granulosa cells are a layer of somatic cells surrounding oocytes, which provide energy for follicular development and oocyte maturation through glycolysis [28]. Studies have shown that the granulosa cell layers around PCOS follicles show atresia, hypertrophy and degeneration [29], which are important causes of PCOS follicle development retardation.

#### 4.1.1. QUR Can Regulate the Sex Hormone Secretion of HPO Axis

There is evidence [30] that treatment with 5, 20 and 45 mg/kg QUR for 50 days can exhibit estrogen-like effects and improve the proportion of various follicles in the ovary of mice, with a decrease in cystic follicles and a significant increase in luteal and normal follicles. Other studies [16,18,21] have found that QUR can reduce luteinizing hormone (LH) and LH/FSH ratio in PCOS rats and improve the expression of E2 and estrogen receptor (ER). However, the regulation of estrogen expression by QUR is still controversial. Contrary evidence [17,19,20] suggests that QUR reduces PCOS estrogen levels. This may be because estrogen is not always expressed too high or too low in PCOS and QUR may regulate estrogen, which is worth further investigation.

#### 4.1.2. QUR Can Inhibit Apoptosis of Granulosa Cells

Naseer Z et al.’s studies [31] found that QUR can reduce the apoptosis of granulosa cells in rabbit follicles and promote the maturation of oocytes in vitro. Granulosa cells are very sensitive to reactive oxygen species (ROS). When the amount of ROS in an ovary is too high, it will lead to oxidative stress and apoptosis of granulosa cells. Two other in vitro studies reported that QUR can reduce the level of ROS, increase the expression of NF-E2-related factor 2(Nrf2) and thioredoxin (Trx) genes and proteins [32] and the activity of antioxidant enzymes [33] and then reduce oxidative stress response and inhibit apoptosis of granulosa cells, which can promote follicular development. As we can see, QUR improves ovulation disorders by reducing the oxidative stress response and protecting granulosa cells.

### 4.2. QUR Can Alleviate IR

Studies have shown that about 50–70% of PCOS patients have IR [34]. Hyperinsulinemia secondary to IR can affect the growth and development of follicles by affecting glucose utilization, decreasing glycolysis and energy supply of follicles [28,29]. Additionally, excess insulin can also increase the level of serum androgens by triggering insulin receptors in the pituitary to release LH and inhibiting the synthesis of sex hormone-binding globulin (SHBG) [35,36]. IR can also increase the risk of lipid metabolism disorders by reducing the expression of lipid droplet proteins in fat cells [37,38]. Therefore, IR is an important pathological hub connecting reproductive and metabolic abnormalities of PCOS.

#### 4.2.1. QUR Activates Insulin Signaling Pathway

Studies [21,24] showed that QUR can reduce serum insulin, resistance hormone and HOMA index in both patients and rat models. These effects of QUR may increase the phosphorylation of insulin receptor substrate 1 (IRS-1) and protein kinase B (AKT), enhancing insulin signal transduction [39,40]. Another study [41] found that QUR can also inhibit the signal transducers and activators of transcription 3 (STAT3)/suppressor of cytokine signaling 3 (SOCS3) signaling pathway to increase hepatic insulin sensitivity.

#### 4.2.2. QUR Can Maintain Glucose Homeostasis

First, QUR can promote glucose utilization by activating the adenine monophosphate-activated protein kinase (AMPK) signaling pathway similar to metformin [42]. AMPK is a key signal-regulating energy metabolism and plays an important role in maintaining energy balance throughout the body. Eid HM et al.’s study showed [43] that QUR can inhibit the activation of AMPK by mitochondrial ATP synthase and increase glucose uptake in muscle cells. Other studies [21,44] reported that QUR activation of the AMPK signaling pathway can stimulate glucose transporter 4 (GLUT4) translocation expression and inhibit G6Pase expression in skeletal muscle to increase glucose uptake. Moreover, Tan Y et al. and Pereira D F et al. [45,46] found that QUR reduces blood glucose levels by inhibiting the activity of α glucoside involved in carbohydrate digestion. These results suggest that QUR can change the disorder of glucose metabolism of PCOS by promoting glucose utilization and decreasing glucose absorption.

#### 4.2.3. QUR Can Protect Pancreatic β Cells

Oxidative stress and inflammation induced by insulin resistance are important causes of pancreatic β cell injury [47], and protecting the function of pancreatic β cells is essential to delay the development of PCOS combined with type 2 diabetes. Youl E et al. [48] found that 20 µmol/L of QUR can activate ERK1/2 to enhance the antioxidant capacity of pancreatic β cells. Additionally, 50 and 100mg/kg of QUR can improve the expression of sirtuin-3 (SIRT3) in type 2 diabetic mice [49] and reduce the levels of nitric oxide (NO) and ROS, thereby reducing the oxidative stress response and preventing the apoptosis of pancreatic β cells. In addition, Dai X et al.’s study [50] showed that QUR can prevent pancreatic β cell death by reducing mitochondrial apoptosis and inhibiting the inflammatory signaling of nuclear factor kappa-B (NF-κB). Iron deposition leads to pancreatic beta cell dysfunction, and iron deposition is present in the serum of PCOS compared to normal subjects [51]. It is reported that QUR inhibits iron deposition in the pancreas and protects pancreatic β cell function in type 2 diabetic mice by eliminating oxidative stress [52]. Moreover, Suganya N et al. [53] found that QUR can improve vascular endothelial function and accelerate β cell recovery by increasing the expression of vascular endothelial growth factor (VEGF) and its receptor VEGFR2 in the pancreas of diabetic rats. Therefore, QUR can protect the function of pancreatic β cells by anti-oxidation, the inhibition of inflammation, reduction in iron death and improvement in pancreatic vascular endothelial function.

### 4.3. QUR Can Reduce the Level of Serum Androgens

Hyperandrogenism is an important phenotype of PCOS, and more than 80% of women presenting with symptoms of androgen excess have PCOS [54]. In PCOS patients, the pituitary is more sensitive to gonadotropin-releasing hormones, leading to increased LH secretion and the induction of androgen synthesis in theca cells. In addition, hyperandrogenism and IR interact as both cause and effect. Excess insulin can promote serum androgen synthesis; increased androgen, in turn, can promote the decomposition of adipose tissue, increase the production of free fatty acids and inflammatory factors and further aggravate IR [55]. Excess androgen can also reduce the sensitivity of granulosa cells to follicle-stimulating hormone (FSH), which leads to follicle development stagnation and a reduced pregnancy rate. In conclusion, hyperandrogenemia is the core lesion of PCOS.

Androgen mainly acts through androgen receptor (AR) binding, and the increased expression of AR is one of the important reasons for the excessive androgen of PCOS. Studies have shown that the AR expression in ovarian granulosa cells of PCOS patients is increased, and the AR alternative splice variant (ASV) with functional disorder exists [56]. Clinical and animal experiments have shown that QUR can reduce androgen levels [16,18,24,26], mainly by inhibiting AR expression and androgen synthesis.

#### 4.3.1. QUR Can Inhibit the Expression of AR

In two in vivo experiments [17,19], QUR inhibits androgen receptor expression in the ovaries of rats with polycystic ovary syndrome induced by dehydroepiandrosterone (DHEA). Zheng S et al. [17] reported that QUR may reduce AR expression by affecting the combination of AR in specific sequences of C-type natriuretic peptide (CNP) and natriuretic peptide receptor 2 (NPR2) gene promoters. In addition, QUR can also increase C-Jun expression in a dose-dependent manner and then inhibit the function and transcriptional activity of the AR promoter [57]. All these indicate that QUR could inhibit the expression of AR.

#### 4.3.2. QUR Can Inhibit Androgen Synthesis

Androgen synthesis mainly comes from the ovary [58]; this process relies on the expression of cytochrome P450 17α-hydroxylase (CYP17A1) and Hydroxysteroid dehydrogenase (HSD), Aldo-keto reductase 1C3 (AKR1C3) and other hormone synthases. Studies have shown that QUR can reduce testosterone concentration in PCOS rats by regulating the expression of the CYP17A1 gene [23] and the activity of 17β-HSD [59]. AKR1C3 is a key enzyme in the final step of testosterone synthesis. Study [60] shows that QUR has a specific ability to inhibit AKR1C3 at low micromolar concentrations and is considered a potential target for the treatment of androgen and other hormone-dependent diseases. In addition, aromatase (CYP19), one of the Cytochrome P450 enzymes, is expressed in granulose cells around developing oocytes in the ovary and is responsible for converting androgens into estrogens. Study [61] shows that QUR is an effective activator of CYP19A1. Mahmoud AA et al. [16] reported that QUR can increase the content of CYP19 in the ovaries of DHEA-induced PCOS rats and reduce the conversion of androgen to estrogen. Above all, QUR can regulate androgen synthase to inhibit androgen synthesis.

### 4.4. QUR Can Improve Abnormal Lipid Metabolism

Epidemiology shows that 38–88% of PCOS in women is combined with overweight or obesity [62]. Fat accumulation leads to active lipolysis, and a large number of fatty acids (FFA) enter the blood, affecting insulin-mediated glucose absorption and promoting the occurrence of IR [63]. In addition, obesity leads to hypertrophy and necrosis of adipocytes and promotes the production of pro-inflammatory cytokines [64]. QUR reduced the weight and body fat percentage of obese women in a randomized, double-blind, placebo-controlled clinical trial [65]. There is also evidence that QUR can reduce serum low-density lipoprotein, triglyceride, cholesterol and other lipid metabolism indexes in obese mice induced by a high-fat diet [66].

#### 4.4.1. QUR Can Promote Fatty Acid Oxidation

Fatty acid oxidation is an important physiological process to maintain the energy balance in the body and occurs mainly in mitochondria [67]. Study [68] showed that QUR can reduce fat formation, accelerate fat decomposition and maintain lipid homeostasis and energy balance in obese mice by activating the AMPK pathway and accelerating fatty acid oxidation. In addition, QUR can also up-regulate the expression of the PPAR-α gene, increase fatty acid oxidation and improve lipid metabolism [69].

#### 4.4.2. QUR Can Regulate Adipokine Production

Adiponectin is one of the main adipokines in the human body, which can improve lipid metabolism, sensitize insulin and fight inflammation [70], and is reduced in PCOS subjects [25,26]. Both clinical trials and animal experiments have shown that QUR supplementation can improve the expression of adiponectin [18,20,24,26] and adiponectin receptors [25] in PCOS, which will be conducive to adiponectin signal transduction and function. Angptl-4 and adipsin are adipokines that promote fat deposition, both of which are elevated in PCOS patients [71,72]. The increase in angptl-4 is mediated by free fatty acids, and the decrease is related to the activation of AMPK pathway [73]. A 20 μM QUR treatment can significantly reduce the levels of angptl-4, adipsin and other adipokines in adipocytes [74]. Therefore, QUR can improve lipid metabolism by regulating adipokine;this has positive meaning for the treatment of PCOS.

#### 4.4.3. QUR Can Induce Browning of Adipose Tissue

Brown adipose tissue (BAT) plays a key role in enhancing metabolism and maintaining energy balance through non-shivering thermogenesis mediated by the expression of the abundant uncoupling protein 1 (UCP1) in mitochondrial intima [75]. Studies found that PCOS patients have a decreased quality and function of brown adipose tissue [63,76], which is a key factor for PCOS, combined with obesity. Feeding 0.05% (*w*/*w*) QUR for 9 weeks can increase WAT Browning and BAT activity through the activation of the AMPK/peroxisome proliferator-activated receptor (PPARγ) pathway in obese mice [77]. Feeding 0.5% onion skin extract increased the expression of brown adipose tissue genes, such as uncoupling protein 1 (UCP1), peroxisome proliferator-activated receptor-γ coactivator-1 (PGC1), CIDEA and TBX1 in obese mice. High-performance liquid chromatography (HPLC) analysis confirmed that quercetin was a functional compound that enhanced the expression of these genes in onion skin extract [78]. The above evidence suggests that quercetin can regulate WAT and BAT ratios and improve fat function to reduce body weight.

### 4.5. QUR Can Regulate Chronic Inflammation

Compared with healthy subjects, the level of serum pro-inflammatory cytokines such as tumor necrosis factor (TNFα) and interleukin-6 (IL-6) were elevated in PCOS patients, presenting a state of chronic inflammation [79,80]. Chronic inflammation can promote apoptosis of granulosa cells, which makes it difficult for PCOS to form dominant follicles. In addition, chronic inflammation can also lead to IR through the induction of pancreatic β cell apoptosis, which indirectly leads to androgen elevation [81]. It is thus obvious that chronic inflammation runs through many pathological links of PCOS.

Interestingly, QUR can directly reduce the level of serum IL-6, TNF-α and other inflammatory factors in PCOS [17,82], reducing inflammation for ovulation disorders, insulin resistance and other adverse effects. Studies have shown that QUR can inhibit the inflammatory response caused by the activation of NLRP3 inflammasome in macrophages [83,84,85]. Other studies confirmed that QUR can regulate the SIRT1/NF-κB signaling pathway to inhibit the transformation and polarization of monocytes into macrophages [86] and improve the inflammatory injury induced by lipopolysaccharide (LPS) [87,88] so as to reduce the secretion of inflammatory factors. In addition, QUR could inhibit the expression of proinflammatory mediators (cyclooxygenase (COX)-1, COX-2) [89,90]. Additionally, Wang et al. [82] found that QUR could inhibit a toll-like receptor (TLR) /NF-κB signaling pathway to improve the inflammatory microenvironment of ovarian tissue, and the results showed that insulin decreased and mature follicles and luteum increased in the PCOS rat model.

### 4.6. QUR Can Regulate Intestinal Flora

The gut contains about 1014 resident microbes [91], of which Firmicutes and Bacteroidetes accounted for more than 90% [92]. Studies [93] showed that in PCOS, the α and β diversity of intestinal flora decreased, and the proportion of Firmicutes and Bacteroidetes was unbalanced. Cani PD et al.’s experimental results [94] showed that the imbalance of intestinal flora could weaken the intestinal epithelial barrier, leading to the entry of LPS into the blood, inducing chronic inflammation and indirectly leading to the occurrence of insulin resistance and obesity. In addition, the dysregulation of intestinal flora can also affect the secretion of neurotransmitters, such as gamma-aminobutyric acid (GABA) in the intestinal brain axis, thus triggering excessive gonadotropin-releasing hormone (GnRH)/LH secretion [95]. Therefore, intestinal flora disorder is an important pathological link in the occurrence and development of PCOS.

Studies show that QUR supplementation significantly increased the relative abundance of Akkermansia and decreased the Firmicutes/Bacteroidetes ratio in obese mice [45,66]. Akkermansia is a Gram-positive strict anaerobic bacterium in the intestinal mucosa, belonging to the Verrucomicrobia, accounting for about 1–3% of the intestinal flora of healthy subjects [96], which can maintain the lipid and glucose homeostasis of the host [97]. In addition, feeding 1% QUR for 16 weeks increased the production of short-chain fatty acids [66]. Short-chain fatty acids are energy-regulating signal molecules generated by carbohydrate metabolism of intestinal flora, which can improve insulin sensitivity [98], down-regulate inflammatory factors [99] and promote fatty acid oxidation [100,101]. Therefore, we believe that QUR can have a positive effect on PCOS by increasing the proportion of beneficial bacteria and the content of short-chain fatty acids.

### 4.7. QUR Can Improve Vascular Endothelial Dysfunction

More and more evidence indicates that PCOS has vascular endothelial dysfunction. The results [102,103] showed that the level of serum NO was decreased, and endothelin-1 (ET-1) and asymmetric dimethylarginine (ADMA) were increased in PCOS patients. By measuring the changes in blood flow resistance in the brachial artery, uterine artery and ovarian artery in PCOS patients, researchers found that PCOS blood flow resistance increased [104,105,106]. All this evidence indicates that PCOS has vascular endothelial dysfunction. The main reason for this phenomenon is the excessive androgen of PCOS, and the high level of androgen binding with the AR in the vascular endothelium can trigger the impaired release of endothelial NO [107], which is an important indicator for the evaluation of vascular dysfunction. In addition, insulin resistance and inflammation are also indirect pathological pathways of PCOS vascular endothelial dysfunction [107,108,109]. Vascular endothelial dysfunction is an early symptom of atherosclerosis, hypertension, diabetes and other diseases secondary to PCOS.

A number of studies have shown that QUR can increase NO, inhibit the expression of endothelin-1, protect the injury of vascular endothelial cells induced by high glucose and restore the normal systolic and diastolic function of vascular endothelial cells [110,111,112]. Chen C et al. [113] reported that long-term oral therapy of QUR protects the vascular endothelium in obese mice by inhibiting PKCδ and the resulting mitochondrial fragments. Chen X et al. [114] found that treatment with 20 μM QUR alleviates the mitochondrial dysfunction of vascular endothelial cells induced by iron deposition through the ROS/ADMA/NO signaling pathway in vitro. In addition, QUR protects vascular endothelial function by inhibiting caveolin-1 phosphorylation and preventing vascular permeability changes under oxidative stress [115]. Unfortunately, QUR has not been reported to directly improve PCOS vascular endothelial function, but it is worthy of further investigation.

## 5. Conclusions

**Figure 2:**
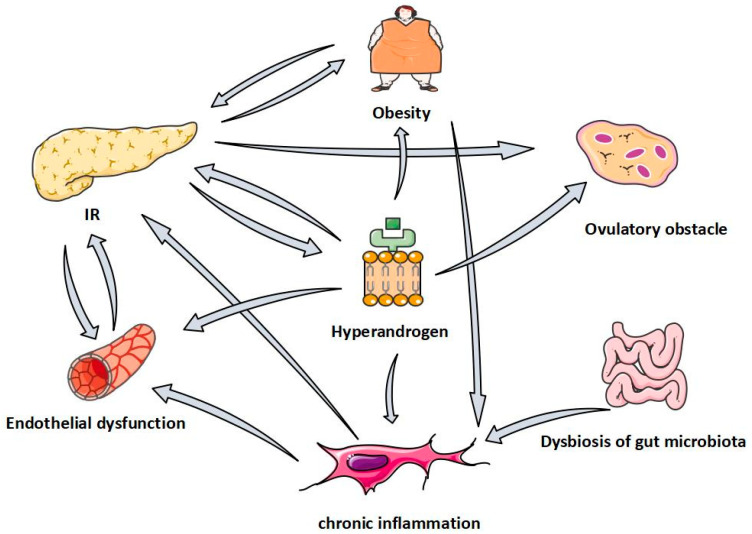
The relationship between pathological links of PCOS.

**Figure 3:**
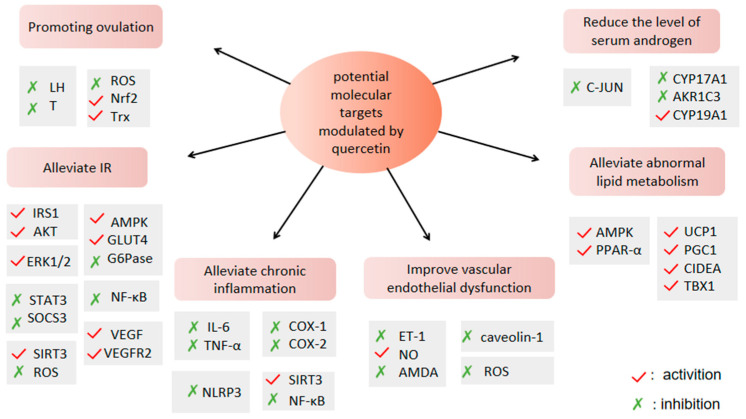
Potential molecular targets of QUR against PCOS.

To sum up, PCOS has pathological links such as ovulation disorders, hyperandrogenemia, insulin resistance, obesity, chronic inflammation, intestinal microflora disorder, vascular endothelial dysfunction and so on, and these pathological links interact and promote each other (Figure 2), exacerbating the occurrence and development of PCOS. QUR plays a potential role in these pathologic aspects of PCOS (Figure 3), and it is not hard to see that QUR works mainly through its antioxidant activity. Of course, this needs to be confirmed in more large-sample, high-level clinical trials. In addition, it is necessary to apply the new technology to improve the bioavailability of QUR in the treatment of PCOS so as to provide more powerful data support for the treatment of PCOS.

## Figures and Tables

**Table 1:** Animal experiments of quercetin in the treatment of polycystic ovary syndrome in the past five years.

Sample Size	Models				Effect of Experimental Group Compared with Control Group									Daily Dosage and Administration of QUR	Time	Reference
		BMI	T	SHBG	LH	LH/FSH	E2	P	HOMA-IR	INS	CHO	TG	PCO			
54	①	**↓**	**↓**	/	**↓**	**↓**	↑	↑	/	/	/	/	improve	25 mg/kg, dissolved in saline, gavage	28 days	[16]
270	①	/	**↓**	/	**↓**	**↓**	**↓**	/	**↓**	/	/	/	improve	100 mg/kg, dissolved in 1% CMC, gavage	28 days	[17]
18	②	-	**↓**	/	/	/	↑	↑	**↓**	**↓**	**↓**	**↓**	improve	100 mg/kg, dissolved in 0.5% CMC, gavage	30 days	[18]
28	①	/	/	/	/	/	**↓**	↑	/	/	/	/	/	100 mg/kg, po	15 days	[19]
/	①	/	/	/	/	/	**↓**	/	/	/	/	/	/	15 mg/kg, dissolved in 10% ethanol, gavage	30 days	[20]
35	①	-	/	/	/	/	/	/	**↓**	**↓**	/	/	improve	15 mg/kg, dissolved in 10% ethanol, gavage	30 days	[21]
24	②	-	**↓**	/	/	/	↑	↑	/	/	**↓**	**↓**	improve	30 mg/kg, po	21 days	[22]
12	③	-	**↓**	/	**↓**	/	/	/	/	**↓**	**↓**	**↓**	improve	150 mg/kg, po	6 weeks	[23]

Experimental group: QUR group; Control group: PCOS model group without intervention. ① Dehydroepiandrosterone (DHEA)-induced PCOS model, ② Letrozole-induced PCOS model, ③ Testosterone propionate-induced PCOS model; **↓**: The experimental group was lower than the control group, and the difference was statistically significant (*p* < 0.05); ↑: The experimental group was higher than the control group, and the difference was statistically significant; -: There were no statistically significant differences between the two groups; /: not mentioned in the study; CMC: carboxy methylcellulose; po: oral administration.

**Table 2:** Clinical experiments of quercetin in the treatment of polycystic ovary syndrome in the past five years.

Sample Size				Effect of Experimental Group Compared with Control Group									Daily Dosage and Administration of QUR	Time	Reference
	BMI	T	SHBG	LH	LH/FSH	E2	P	HOMA-IR	INS	CHO	TG	PCO			
80	**↓**	**↓**	↑	**↓**	/	/	/	**↓**	**↓**	/	/	/	1000 mg, capsules, po	12 weeks	[24]
84	-	**↓**	-	**↓**	/	/	/	**↓**	**↓**	/	/	/	1000 mg, capsules, po	12 weeks	[25,26]

**↓**: The experimental group was lower than the control group, and the difference was statistically significant (*p* < 0.05); ↑: The experimental group was higher than the control group, and the difference was statistically significant; -: There were no statistically significant differences between the two groups; /: not mentioned in the study.

## Data Availability

Not applicable.
